# Evaluation of Caspase-9b and PP2Acα2 as potential biomarkers for chronic lymphocytic leukemia

**DOI:** 10.1186/s40364-016-0063-6

**Published:** 2016-05-04

**Authors:** Leticia Domínguez-Berrocal, Xiguang Zhang, Jean Marc Zini, Jesús Fominaya, Angelita Rebollo, Jerónimo Bravo

**Affiliations:** Instituto de Biomedicina de Valencia, Consejo Superior de Investigaciones Científicas (IBV-CSIC), c/ Jaime Roig 11, 46010 Valencia, Spain; Institut National de la Santé et de la Recherche Médicale (INSERM), U1135, 83, Université Pierre et Marie Curie, 91 bd de l’Hôpital, 75013 Paris, France; Assistance Publique – Hôpitaux de Paris (AP-HP), Hôpital Saint Louis, 1 avenue Claude Vellefaux, 75010 Paris, France

**Keywords:** Caspase-9, Caspase-9b, PP2Acα, PP2Acα2, Chronic lymphocytic leukemia, Alternative splicing, Biomarker

## Abstract

**Background:**

Disruption of alternative splicing in apoptotic factors has been associated to chronic lymphocytic leukemia among other cancers and hematological malignancies. The proapoptotic proteins Caspase-9 and PP2Acα are functionally related in a direct interaction, which constitutes a promising target for cancer therapy. Both proteins present aberrant mRNA splicing variants that are antiapoptotic (Caspase-9b) and catalytically inactive (PP2Acα2), respectively.

**Results:**

In this work we have analyzed the relative abundance of the aberrant spliced forms Caspase-9b and PP2Acα2 in several cell lines and chronic lymphocytic leukemia patients and correlated it with several parameters of the disease. Despite 40 % of the patients presented Caspase-9b dysregulation, there was no direct association between alterations in Caspase-9b relative abundance and the parameters analyzed in medical records. More importantly, PP2Acα2 dysregulation was observed in 88 % of CLL patients and was related with advanced stages of the malignancy.

**Conclusions:**

Caspase-9b dysregulation seemed to be associated with the disease, although the differences between healthy donors and CLL patients were not statistically significant. However, PP2Acα2 dysregulation was significantly different between healthy donors and CLL patients and correlated with Binet B and C stages; therefore, we propose the use of PP2Acα2 dysregulation as a potential biomarker for advanced stages of chronic lymphocytic leukemia.

## Introduction

Chronic lymphocytic leukemia (CLL) is the most common B-cell malignancy in Caucasian aging adults, rarely younger than 50 years old [1]. Disruption of alternative splicing in many apoptotic factors is related to hematological malignancies and cancer, as CLL [2–6]. Abnormally expressed splicing factors in tumor cells induce the production of mRNA isoforms that are nonexistent or less abundant in normal cells, thus contributing to cancer development, tumor progression, different response to therapy and chemorefractoriness [7, 8].

Caspase-9 is a key point in the apoptotic signal transduction. The expression of its mRNA spliced variant Caspase-9b, lacking exons 3 to 6, inhibits apoptosis in a dominant-negative manner [9], which may establish a threshold to regulate Caspase-9 activation and prevent undesired apoptosis [10]. Caspase-9b is dysregulated in astrocytoma [11] and in several subtypes of non-small-cell lung cancer (NSCLC). Moreover, its overexpression was responsible of maintaining the tumorigenic capacity of NSCLC cells and made cells resistant to erlotinib [12]. Targeting the alternative splicing of Caspase-9 sensitized NSCLC cells to chemotherapies, increasing their efficiency and limiting their toxic side-effects [13].

The direct interaction between Caspase-9 and PP2Acα has been previously described [14]. PP2A is one of the major Ser/Thr phosphatases, whose dysregulation is associated with multiple cancers among other functions [15]. A catalytically inactive aberrant isoform of PP2A catalytic C subunit, PP2Acα2, has been reported, missing exon 5, which is close to the active site. PP2Acα2 has only been observed overexpressed in peripheral blood mononuclear cells (PBMC) under starvation conditions but the ratio PP2Acα:PP2Acα2 was reestablished once they were transferred to culture medium. When analyzed in cell lines, PP2Acα2 was present either as mRNA or as protein but at almost undetectable levels [16].

The aim of the present work was to study the expression ratios Caspase-9: Caspase-9b and PP2Acα:PP2Acα2 in cell lines and more importantly in healthy donors and CLL patients to evaluate their association with the disease.

## Material and methods

### Cell lines culture

HeLa cells were cultured in DMEM + 10 % FBS, Daudi and Jurkat cells in RPMI 1640 + 10 % FBS and SH-SY5Y in DMEM F12 + 10 % FBS, 1 % Glutamine and 1 % Hepes 1 M. All cell lines were grown at 37 °C and 5 % CO_2_ and underwent passage three times a week.

### B cells isolation

Fresh blood from healthy donors was obtained from the Établissement français du sang. CLL samples were obtained from the Hematology Department of Saint Louis hospital (Paris). PBMC were isolated by Ficoll gradient centrifugation for 20 min at 2300 rpm, they were collected and washed twice with PBS. B cells were isolated using DynaI negative isolation kit (Invitrogen), reaching around 98 % purity.

### Conventional PCR and Real Time PCR

Total RNA was extracted with TRIzol® (Life Technologies), cDNA was obtained performing RT-PCR using High Capacity cDNA Reverse Transcription Kit (Applied Biosystems) and conventional PCR was set up with Kapa HiFi DNA polymerase (Kapa Biosystems) at 95 °C-3’, 30 cycles of 98 °C-20”, 55 °C-20”, 72 °C-1’30” and a final extension of 72 °C-5’. The primers used were FWD: 5’-ATGGACGAAGCGGATCGG-3’ and REV: 5’-TTATGATGTTTTAAAGAAAAGTT-3’ for Caspase-9 and FWD 5’-GACGAGAAGGTGTTCACCAA-3’ and REV 5’-TTACAGGAAGTAGTCTGGGGTAC-3’ for PP2Acα.

Real Time PCR was performed using TaqMan PCR Mastermix in a 7500 Fast Real Time Applied Biosystems device. Caspase9 and PP2Acα probes corresponded to Hs00154261_m1 and Hs01003394_mH Life Technologies references. Caspase9b probe was previously described [17] and PP2Acα2 primers and probe were designed: 5’-CAAGAAGTTCCCCATGAGGGATATA-3’ (forward), 5’-CAACGATAACAATAGTTTGGAGCACT-3’ (reverse) and 5’-CGTTACTACATTCCGGTCATGGCACCA-3’ (probe). GAPDH was used as housekeeping reference gene (Hs99999905_m1, Life Technologies). Data analysis of relative expression was calculated following the 2^-ΔΔC^_T_ method [18]. Differences between healthy donors and CLL patients were statistically evaluated with Student's *t* test and with a Two-way ANOVA with replication with a significance level of *P* < 0.005.

## Results and discussion

### Caspase-9b and PP2Acα2 expression in cell lines

**Fig. 1:**
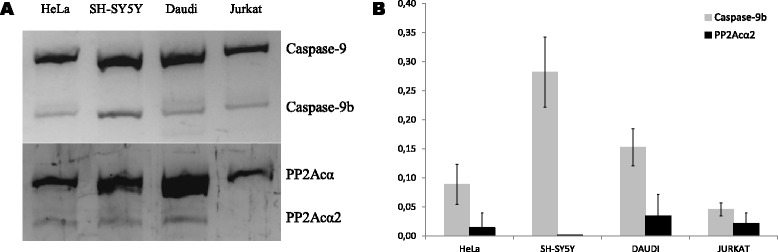
Analysis of Caspase-9b and PP2Acα2 in several cancer cell lines. **a**. Conventional PCR analysis of cancer cell lines showing Caspase-9 and PP2Acα full length and spliced variants expression. **b**. Real Time PCR of cancer cell lines. Caspase-9b and PP2Acα2 relative abundance is represented in the graph and normalized with respect to Caspase-9 and PP2Acα respectively, considered as 1

Among the cell lines analyzed, derived from different oncologic malignancies, all of them showed a healthy ratio of Caspase-9b relative abundance in PCR and Real Time PCR assays (Fig. 1a and b). As established by Shultz et al. [19], the criteria used to classify Caspase-9b levels were: normal (Caspase-9: Caspase-9b mRNA ratio ≥3.3), moderately dysregulated (3.3 > X > 2.2) and highly dysregulated (≤2.2). Following the criteria that we established for CLL patients as described below, cell lines possessed PP2Acα2 mRNA levels corresponding to healthy state, obtaining very low values in Real Time PCR and a faint band in conventional PCR, when detected (Fig. 1a and b). These results suggest that Caspase-9b or PP2Acα2 dysregulation wouldn’t be characteristic of the analyzed cell lines, which prompted us to consider the approach of using samples from CLL patients instead of insisting with other established cell lines. Patients samples of these pathologies should be analyzed to confirm cell lines data; nevertheless in previous studies Caspase-9b dysregulation has been observed in the same extent in NSCLC cells and patients [12].

### Caspase-9b and PP2Acα2 expression in healthy donors and CLL patients

**Fig. 2:**
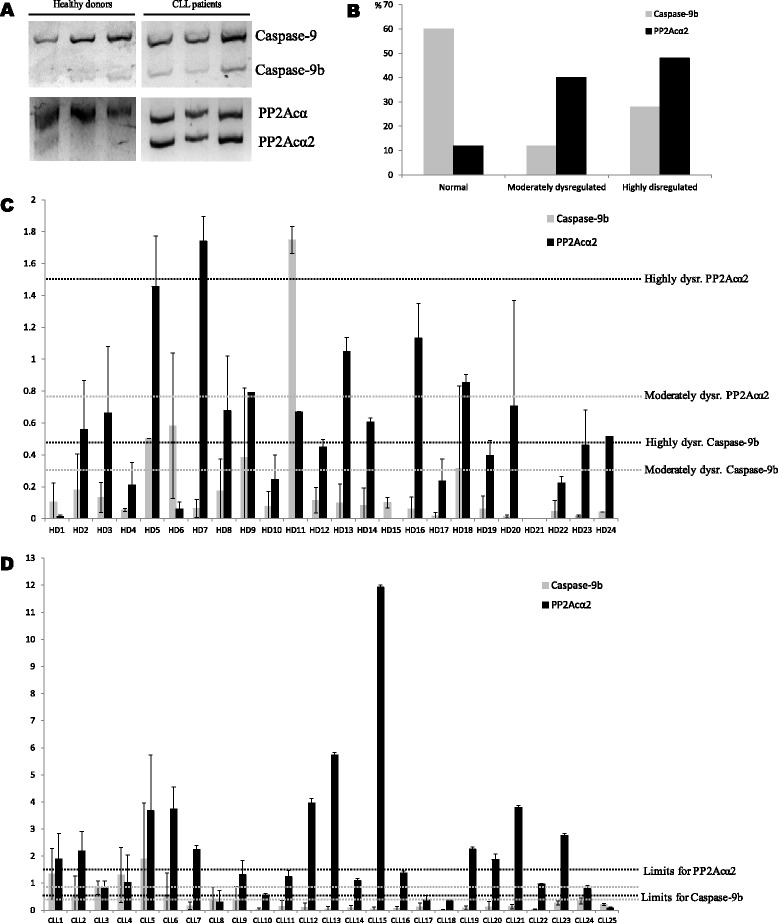
Analysis of Caspase-9b and PP2Acα2 in healthy donors and CLL patients. **a**. Conventional PCR analysis of three healthy donors and three CLL patients for Caspase-9 and PP2Acα full length and spliced variants expression. **b**. Percentage of CLL patients that present normal, moderately dysregulated and highly dysregulated relative abundance of Caspase-9b and PP2Acα2. **c**. Real Time PCR analysis of healthy donors. Relative abundance values of spliced forms have been normalized with respect to full length forms, considered as 1. The limits for moderately dysregulated values are marked with a grey dotted line and the limits for highly dysregulated values with a black dotted line. **d**. Real Time PCR analysis of CLL patients. The same criteria as for Fig. 2c were applied for calculations and limits representation. Asterisk represents *P* < 0.005 (*P* = 0.0024), when compared with healthy donor

Conventional PCR showed that Caspase-9b was slightly expressed in healthy donors, while CLL patients showed a moderate overexpression. Healthy donors showed no detectable or very low expression of PP2Acα2, but the majority of CLL patients presented from moderate to high overexpression of the aberrant form (Fig. 2a).

A cohort of 24 healthy donors and 25 CLL patients (15 men and 10 women) was analyzed by Real Time PCR. Figure 2b summarizes Caspase-9b and PP2Acα2 expression in CLL patients. 80 % of healthy donors showed Caspase-9b normal ratio and the rest were slightly over healthy values (Fig. 2c). Unlike in NSCLC [19], where 36 % of the patients were moderately dysregulated and 42 % were highly dysregulated, in CLL only 40 % of the patients had some alteration in Caspase-9b expression (Fig. 2d), although the highly dysregulated also overcame the moderate percentage (28 % versus 12 %).

According to Shultz et al.[19] criteria for Caspase-9b dysregulation, 80 % of the healthy donors that we analyzed fitted into normal values for Caspase-9b. However, PP2Acα2 was expressed in healthy donors in a PP2Acα:PP2Acα2 ratio from negligible values up to 1.27 in 80 % of the samples (Fig. 2c). Therefore, we formulated classification criteria that would fit a similar percentage of healthy donors in each category as the ones described by Schultz for Caspase-9b. We established a mRNA ratio PP2Acα:PP2Acα2 ≥ 1.33 for healthy expression, 0.67 < X < 1.33 for moderately dysregulated and ≤ 0.67 for highly dysregulated. According to this, 88 % of CLL patients analyzed had overexpression levels of PP2Acα2 (Figs. 2b and d). The efficacy of the use of PP2Acα2 as a CLL biomarker and the applicability of our criteria was validated with the calculation of several clinical parameters. The prevalence of the disease in the whole cohort was 51.02 % and the use of PP2Acα2 as a biomarker presented a sensitivity of 78.57 %, a specificity of 85.71 %, a positive predictive value of 88 % and a negative predictive value of 75 %. According to these results, the detection of a PP2Acα2 overexpression in a patient will allow to diagnose CLL in a 78.57 % of the cases. The specificity of this biomarker assures that 85.71 % of the patients without a dysregulation of PP2Acα2 don’t present the disease and only a 14.29 % would be diagnosed as false positives.

**Fig. 3:**
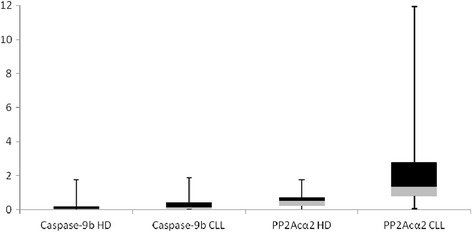
Normalized relative abundance of Caspase-9b and PP2Acα2 in healthy donors and CLL patients populations. Second quartile is represented in grey and third quartile in black. Maximums and minimums are shown with error bars

Gathering the data of relative abundance of spliced variants with the medical records available [20, 21] (Table 1), we couldn’t establish a direct correlation between the dysregulation in Caspase-9b and any of the disease parameters analyzed, but we don’t exclude that it may be related to other features of this malignancy. However, this dysregulation in 40 % of CLL patients makes us consider it as a manifestation of the disease and it could be a potential biomarker in some extent, although the differences between healthy donors and CLL patients were not statistically significant according to Student’s *t* test (*P* = 0.1878). Interestingly, all individuals classified in the medical records as B or C Binet score [20] presented highly dysregulated PP2Acα2, being the only patient with score C the one with the highest PP2Acα2 levels. Among A score patients, PP2Acα2 levels from normal to highly dysregulated were found. In this case, the differences between healthy donors and CLL patients for PP2Acα2 relative abundance were statistically significant (*P* < 0.005, *P* = 0.0024). A two-way ANOVA with replication was also performed between healthy donors and CLL patients 1–24 for Caspase-9b and PP2Acα2 relative abundance. The differences between healthy donors and CLL patients were significant (*P* < 0.0005) and also between Caspase-9b and PP2Acα2 (*P* < 0.00005). The interaction P-value (*P* < 0.005) suggested that the variables Caspase-9b and PP2Acα2 are different depending on the level of the other variable respectively. The distribution of Caspase-9b and PP2Acα2 relative abundance in the healthy donors and CLL patients populations was represented in Fig. 3. Although more extensive studies should be performed to confirm these data, we have promising evidences that suggest the importance of PP2Acα2 in the course of CLL. For the first time, the aberrant spliced variant PP2Acα2 has been related with a pathological state, suggesting its implication in the disease and a possible function for a protein that remains known as inactive.

**Table 1:** Available medical records of the CLL patients analyzed

Patient	Age at diagnosis	Previous treatments	White blood cells	Hemoglobin	Platelets	Matutes score	Binet score	Caspase-9b	PP2Acα2
CLL15	71	NO	100000	10.2	86	4	C	0.05	11.93
CLL13	56	NO	97000	11.7	230	5	B	0.05	5.73
CLL12	63	NO	85000	12.7	116	5	B	0.10	3.96
CLL21	64	NO	110000	9.8	95	5	B	0.13	3.80
CLL6	82	NO	28000	10.6	206	5	B	0.57	3.74
CLL19	60	YES	230000	12.5	90	5	B	0.09	2.26
CLL5	72	NO	200000	12.9	92	5	A	1.90	3.68
CLL2	78	NO	100000	12.3	223	4	A	0.55	2.19
CLL1	50	NO	130000	10.9	189	5	A	1.34	1.89
CLL20	68	NO	87000	10.6	120	5	A	0.12	1.87
CLL16	43	NO	44000	15.0	210	5	A	0.06	1.38
CLL11	75	NO	25000	14.2	186	4	A	0.14	1.24
CLL4	72	NO	27000	14.6	250	5	A	1.30	1.02
CLL3	68	NO	80000	13.1	188	5	A	0.83	0.82
CLL17	87	NO	140000	10.1	66	5	A	0.11	0.38

Age, treatment with fludarabin (in the case of CLL19), hematological parameters and Matutes score (that confirms these patients as CLL) registered in medical records for some of the patients analyzed are compiled in this table. Binet score B and C, indicating advanced stages of the disease, are highlighted in bold. Normalized values of relative abundance of Caspase-9b and PP2Acα2 have been added to the table

Several markers as CD38, ZAP-70 and IgV_H_ mutation status among others have been implemented as CLL progression and diagnosis predictors not without certain controversy [22–24]; therefore we propose also the use of PP2Acα2 high dysregulation as a potential CLL biomarker associated to severe stages of the disease.

Disruption of the PP2Acα:PP2Acα2 ratio in CLL patients may also alter the equilibrium of Caspase-9/PP2Acα interaction, giving rise to aberrant Caspase-9/PP2Acα2 complexes. Interestingly, we have previously reported a bifunctional peptide termed DPT-C9h, capable of dissociating Caspase-9/PP2Acα complex [14]. Given that Caspase-9 binding site is present in both PP2Acα and PP2Acα2, DPT-C9h may also interfere in the hypothetical Caspase-9/PP2Acα2 interaction, contributing to a possible modulation of the DPT-C9h mechanism of action and differential treatment response [14].

This bifunctional peptide induced apoptosis in CLL B cells without affecting healthy B cells nor the rest of peripheral blood mononuclear cells [25]. The bifunctional peptide DPT-C9h was also capable of inducing apoptosis in several cancer cell lines and in tumor xenograft models with treatment response in a different extent, which may be related to the relative abundance of PP2Acα2 in the cell lines and patients tested [14]. Further experiments will be performed to shed light on the role of PP2Acα2 in CLL and the hypothetical differential response to DPT-C9h depending on the level of PP2Acα2 dysregulation.

Caspase-9b has been related to disease state in astrocytoma [11] and to play an important role in NSCLC treatment response [12]. Our results provide first evidences for the presence of aberrant PP2Acα/PP2Acα2 ratios in CLL advanced stage patients. Therefore, the dysregulation of the splicing variants of the association Caspase-9/PP2Acα is emerging as a valuable tool as biomarkers for prognosis in cancers that present aberrant expression of Caspase-9b or PP2Acα2 spliced variants. Further studies will need to be performed to evaluate Caspase-9/Caspase-9b and PP2Acα/PP2Acα2 ratios in other types of cancer and their possible application as biomarkers.

### Ethics approval and consent to participate

The experiments with human samples included in this work were performed in accordance with the Declaration of Helsinki. No ethics committee approval was required for these experiments. Written informed consent was obtained from all patients.

## Abbreviations

CLL: chronic lymphocytic leukemia
DMEM: Dulbecco’s modified Eagle medium
DPT: drug phosphatase technology
FBS: fetal bovine serum
NSCLC: non-small-cell lung cancer
PBMC: peripheral blood mononuclear cells
RT-PCR: reverse transcription polymerase chain reaction
